# Research on High-Value Utilization of Carbon Derived from Tobacco Waste in Supercapacitors

**DOI:** 10.3390/ma14071714

**Published:** 2021-03-31

**Authors:** Zhenrui Huang, Caiyun Qin, Jun Wang, Lin Cao, Zhuwen Ma, Qinghua Yuan, Zhidan Lin, Peng Zhang

**Affiliations:** 1 Guangdong Provincial Engineering & Technology Research Center for Tobacco Breeding and Comprehensive Utilization, Crops Research Institute Guangdong Academy of Agricultural Sciences, Guangdong Provincial Key Laboratory of Crop Genetics and Improvement, Guangzhou 510640, China; fjsi@163.com (Z.H.); conghuama@163.com (Z.M.); qinghuay@foxmail.com (Q.Y.); 2 Institute of Advanced Wear & Corrosion Resistant and Functional Materials, Jinan University, Guangzhou 510632, China; CaiyunQin777@163.com (C.Q.); linc19993@163.com (L.C.); 3 Nanxiong Institute of Tobacco Research, Nanxiong 512400, China; wangjun4170@126.com

**Keywords:** tobacco stalk, biomass carbon, hydrothermal, supercapacitor

## Abstract

Large quantities of tobacco stalks residues are generated and discarded as crop waste or combusted directly every year. Thus, we need to find an appropriate way to dispose of this type of waste and recycle it. The conversion of biomass waste into electrode materials for supercapacitors is entirely in line with the concept of sustainability and green. In this paper, tobacco-stalk-based, porous activated carbon (TC) was successfully synthesized by high-temperature and high-pressure hydrothermal pre-carbonization and KOH activation. The synthesized TC had a high pore volume and a large surface area of 1875.5 m^2^ g^−1^, in which there were many mesopores and interconnected micro-/macropores. The electrochemical test demonstrated that TC-1 could reach a high specific capacitance of up to 356.4 F g^−1^ at a current density of 0.5 A g^−1^, which was carried in 6M KOH. Additionally, a symmetrical supercapacitor device was fabricated by using TC-1 as the electrode, which delivered a high energy density up to 10.4 Wh kg^−1^ at a power density of 300 W kg^−1^, and excellent long-term cycling stability (92.8% of the initial capacitance retention rate after 5000 cycles). Therefore, TC-1 is considered to be a promising candidate for high-performance supercapacitor electrode materials and is a good choice for converting tobacco biomass waste into a resource.

## 1. Introduction

The rapid increase in the volume of waste agricultural biomass, as a result of intensive agriculture in the wake of population growth and worsening environmental pollution, is becoming a burgeoning problem because rotten waste agricultural biomass depletes soil nutrients by taking up more nitrogen, phosphorus, and potassium, and open burning by farmers to clear their lands generates CO_2_ and other local pollutants [1]. Tobacco stalks as crop waste contain a large number of bacteria; directly discarding or returning them to farmland can easily transmit and spread disease, including anthracnose and orange disease. Open-air combustion could pollute the atmosphere and cause the waste of resources and pollution to varying degrees. Therefore, how to recover tobacco stalks with high efficiency and high value has a certain practical significance. A variety of biomass or biological wastes, such as corn cob [2], orange peel [3], rice husks [4], goldenberry calyx [5], teak [6], wheat straw [7], shiitake substrate [8], waste tea [9], and jute fiber [10], were used to produce activated carbon materials.

It is worth noting that porous activated carbon is an ideal electrode material for supercapacitors. Biomass-derived porous carbon can inherit special microstructures from precursors, which provide active sites for many ions transport channels and energy storage. High specific surface area and hierarchical porous structure are the most important two main factors, which would affect the electrochemical properties of carbon materials [11]. A high specific surface area could provide sufficient active positions for charge storage. The appropriate pore structure can improve capacitance by increasing the electrode–electrolyte interface area and providing a suitable channel for electrolyte ions’ entry [12]. In previous studies, most of the porous carbons were synthesized by a two-step method [13,14,15,16]. The first step was pre-carbonization, and the second step was pyrolysis or activation. There have been many previous studies that preferred low-temperature pyrolysis pre-carbonization. Hydrothermal carbonization (HTC) greatly impacts the synthesis of hydrocarbon, and hydrothermal carbonization can transfer oxygen-rich substituents to carbon materials [17]. HTC technology is also a simple, economical, and efficient method [18]. It is one of the most popular thermochemical conversion techniques for biomass carbon-based materials in terms of preparation strategy, with two advantages. First, because of hydrothermal carbonization and chemical activation, the subsequent activation process’s efficiency is improved, reducing the total cost of production. Second, the two-step process improves the microstructural characteristics of the final material [19].

Appropriate specific surface area and pore size distribution are the key factors affecting the capacitive properties of carbon materials. Physical activation and chemical activation are usually used to improve the specific surface area of carbon materials. Chemical activation has attracted attention due to its high yield, low cost, short activation time, and low temperature to form porous structures. Commonly used activators are KOH [20], NaOH [21], ZnCl_2_ [22], etc. Compared with other activators, KOH is the most effective and environmentally friendly activator; therefore, it is more preferred to prepare porous activated carbon in chemical activation processes [23]. Xia et al. [24] used a simple KOH activation method to prepare tobacco stem-based activated carbon and successfully used it as an electrode in electric double-layer capacitors. The specific capacitance reached 190 F g^−1^ at a current density of 1 A g^−1^. Xue et al. [25] used tobacco stems as carbon precursors to prepare microporous and mesoporous carbon, and as negative electrode material for lithium–ion batteries to obtain a high capacity of 1689 mAh g^−1^ at 500 mA g^−1^. Mudyawabikwa et al. [26] used microwave heating to prepare activated carbon from tobacco stems, in which the activated carbon was used as an adsorbent to remove methylene blue (MB) from water. A previous study of our research group [27] showed that the activated carbon prepared by KOH activation of tobacco stalks has a specific capacitance of 281.3 F g^−1^ at a current density of 1 A g^−1^. Afterward, the activated carbon was nitrogen–sulfur doped and the specific capacitance was increased to 422.5 F g^−1^.

This study provides a much higher pressure to synthesized hierarchical pores for preparing activated carbon from tobacco stalks. In the first stage, the tobacco stalk was pretreated by a high-temperature, high-pressure hydrothermal method with a high-pressure reactor. The HTC pressure depends on the temperature. Higher HTC reaction temperature and pressure increases the pore volume, decreases the pore radius, increases the material’s conversion rate, and removes the ash content of the material, thus improving the subsequent activation efficiency. In this study, hydrothermal carbon with a high degree of aromatization and a large number of oxygen-containing groups was used. Then, KOH was used as an activator to adjust the surface chemical composition and pores of the material. Most papers dealing with pre-carbonization use an imposed pressure. However, when the only known parameter is the temperature, the consideration of pore synthesis for enhancing the surface area is mandatory. Therefore, a certain high pressure in this study was used to determine whether pressure can improve the surface roughness of the material, increasing the effective specific surface area.

## 2. Materials and Methods

### 2.1. Materials

Tobacco stalk was obtained from Crops Research Institute, Guangdong Academy of Agricultural Sciences. Potassium hydroxide (KOH, Damao chemical reagent factory, Tianjing, China), analytical grade, hydrochloric acid (HCl, Guangzhou Chemical Reagent Factory, Guangzhou, China), analytical grade, and deionized water were utilized throughout the experiments. All reagents were used without further purification.

### 2.2. Synthesis of Tobacco Stalk-Derived Hierarchical Porous Carbon

**Figure 1:**
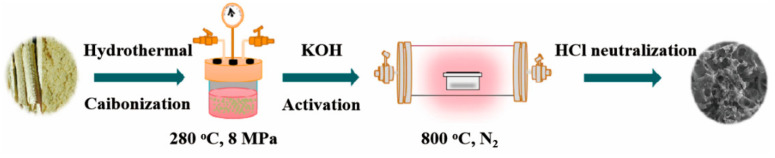
Schematic synthesis of tobacco-stalk-based hierarchical porous carbon (TC).

Hierarchical porous carbon was prepared by KOH activation utilizing tobacco stalk as the carbon precursor. The schematic synthesis of tobacco stalk-based hierarchical porous carbon is shown in Figure 1. Firstly, the tobacco stalk was dried at 90 °C for 24 h after repeatedly washing with deionized water, and then a planetary type ball mill was used to ground the stalk into a powder and sieve it through a 200 mesh. The ball-to-tobacco stalk ratio was kept at 10:1 and milling speed was adjusted to 400 rpm. In order to avoid excessive heat generation and prevent powder from adhering to the walls and steel balls, the rotation direction of the rolling mill was reversed at constant intervals and adjusted to 300 rpm. Next, powder and water were put into a stainless-steel reactor at a volume ratio of 1:5 for 12 h (280 °C, 8 MPa) hydrothermal treatment, during which the hydrothermal carbon (HC) was transformed. The HC samples were activated with KOH at the different mass ratios in the N_2_ atmosphere at 800 °C for 2 h. Then, they were washed with 1 M HCl and deionized water until neutral. Finally, tobacco-stalk-based, porous activated carbon (TC) was obtained by being dried at 90 °C for 24 h. HC samples activated by different KOH mass ratios (KOH:HC = 0:1, 0.5:1, 1:1, 2:1) were labeled TC-0, TC-0.5, TC-1, and TC-2, respectively (as shown in Table 1). We also prepared a contrast sample, i.e., the sample that was not hydrothermally pre-carbonized, nor mixed with KOH, directly pyrolyzed at 800 °C and named Blank-TC.

### 2.3. Characterization

The morphology and structure of the TC were characterized by field-emission scanning electron microscopy (FESEM, Ultra55, ZEISS, Jena, Germany) and field-emission transmission (TEM, JEOL JEM-2100F, Tokyo, Japan). To confirm the possible phase and composition of the sample further, X-ray photoelectron spectroscopy (XPS, Escalab 250Xi, UK) with monochromatic AlKα radiation source at room temperature and Raman spectrometers (LR-3, Varian, Palo Alto, CA, USA) with a 633-nm laser light were used for characterization. Moreover, the BeiShiDe 3H-2000PS2 (Beijing, China) surface area porosity analyzer was used to obtain the pore-size distribution and apparent surface area of the sample through the nitrogen adsorption/desorption isotherm. The Brunauer–Emmett–Teller (BET) specific surface areas, Barrett–Joyner–Halender (BJH) pore-size distribution of TC were obtained from the Nitrogen-adsorption/desorption isotherms recorded at 77 K (ASPA2460, Micromeritics, Shanghai, China).

### 2.4. Electrochemical Measurements

The galvanostatic charge–discharge (GCD), cyclic voltammetry (CV), and electrochemical impedance spectroscopy (EIS) of the TC samples in this paper were tested in a CHI760E workstation (CH Instruments, Austin, TX, USA) using 6 M KOH. A saturated calomel electrode served as the reference electrode, and a platinum electrode served as the counter electrode. The working electrode was prepared by mixing active material with TC, acetylene black, and polytetrafluoroethylene (PTFE) at a mass ratio of 80:10:10, coating on a nickel foam sheet (1 * 2 cm^2^). The active material mass of TC-0, TC-0.5, TC-1, and TC-2 were 3.2, 3.2, 1.6, and 2.4 mg. The CV curves were tested at 10–200 mV s^−1^ in a potential window of −1.0–0 V. The GCD curves were measured at different current densities (0.5–10 A g^−1^), and the EIS was performed within a frequency range of 100 kHz–0.01 Hz. Formula (1) was used to calculate the specific capacitance (*Cs*) from the GCD curve as follows:

(1) $$C_{s}=\frac{I\Delta t}{m\Delta V}$$

where *C_s_*(F g^−1^), *I*(A), ∆*t*(s), m(g), and ∆*V*(V) represent specific gravity capacitance, current density, discharge time, active material mass, and potential range, respectively.

Sequentially, the symmetric supercapacitor was assembled in a CR2032 stainless steel coin cell. We coated TC-1 on a 1.5 cm diameter foamed nickel sheet as the positive and negative electrodes, and the mass of TC-1 on the two electrodes was the same (8.64 mg), using the 6-M KOH as the electrolyte and the polypropylene membrane as the separator. Then, we tested its electrochemical performance (CV, GCD, EIS). The specific capacitance (*C_m_*, F g^−1^), energy density (*E*, Wh kg^−1^), and power density (*P*, W kg^−1^) of the symmetric capacitor were calculated as follows:

(2) $$C_{m}=4C_{cell}=\frac{4I\Delta t}{m\Delta V}$$

(3) $$E=\frac{4C_{cell}\Delta V^{2}}{2\times 3.6}$$

(4) $$P=\frac{3600E}{\Delta t}$$

where *C_cell_* is the specific capacitance of symmetric supercapacitors, *C_m_* is the specific capacitance of a single electrode, *I*(A) is the current density, ∆*t*(s) is the discharge time, *m*(g) is the total mass of active material, and ∆*V*(V) is the potential range [28].

## 3. Results and Discussion

**Figure 2:**
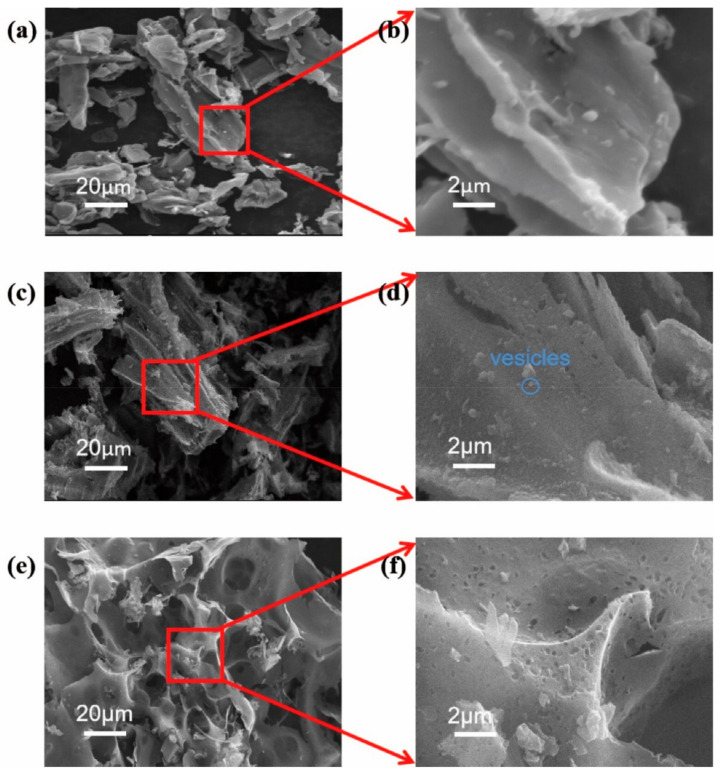
Field-emission scanning electron microscopy (FESEM) images of (**a**,**b**) Blank-TC, (**c**,**d**)TC-0 and (**e**,**f**) TC-1 samples at various magnifications.

According to literature results [29], higher temperature and higher pressure are the key points for pre-carbonization during the hydrothermal process. The decomposition of cellulose and lignin during hydrothermal processes leads to increased surface roughness and vesicle production, providing more possibilities for the next activation. A high-pressure reactor was adopted to pre-carbonize tobacco waste under 280 °C and 8 MPa and then was activated with KOH at different mass ratios (1:0, 1:0.5, 1:1, 1:2) in the N_2_ atmosphere at 800 °C for 2 h. The morphology of different tobacco carbon species is shown in Figure 2. The sample was directly pyrolyzed to 800 °C without hydrothermal pre-carbonization and is shown in Figure 2a,b. The relatively smooth surface of Blank-TC was observed. In comparison to Blank-TC, the TC-0 sample showed a rough surface and few vesicles (Figure 2d). Moreover, an apparent porous nanostructure was observed in the micrograph of TC-1 (Figure 2f). The rough surface and porous structure facilitate the contact area of the active material with the electrolyte. It increases the density of the active center and promotes ion transport. After combined HC and KOH activation, the material had a high specific surface area and abundant pores, optimizing the electrochemical properties. The waste tobacco stalk material could be effectively applied to the negative electrode material of the supercapacitor.

**Figure 3:**
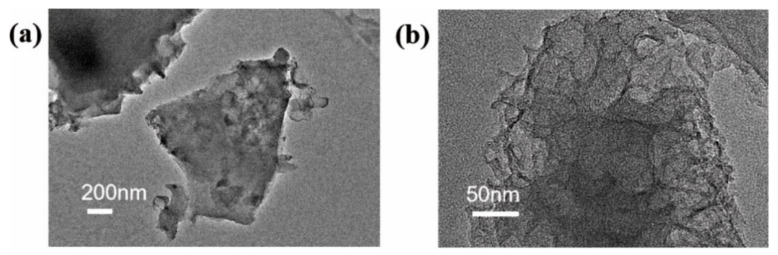
TEM images of TC-1 at (**a**) low magnification and (**b**) high magnification.

The pore structure of TC-1 was further observed by TEM (Figure 3). TC-1 exhibited an interconnected porous structure. Abundant micropores and mesopores of the carbon skeleton were observed from high-resolution TEM images. The interconnected porous structure act as electrolyte reservoirs to ensure a rapid entry of ions into the porous carbon wall, facilitating rapid electron transport and charge storage. These advantages result in a structure with electrochemical properties.

**Figure 4:**
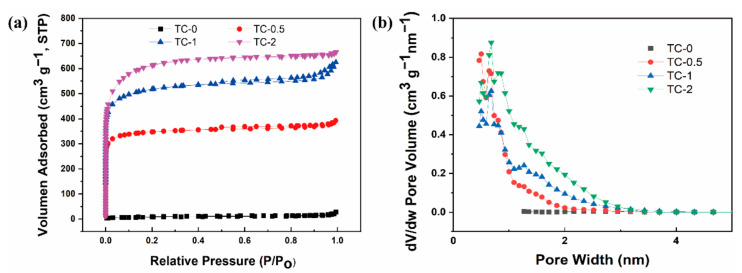
(**a**) Nitrogen−adsorption/desorption isotherms at 77 K, and (**b**) Barrett–Joyner–Halender (BJH) pore−size distributions of four samples.

The surface area and porous structure of carbon materials were measured by nitrogen adsorption/desorption. Figure 4a shows the isotherms of all samples. All isotherms had a similar shape, with a mixture of Type I and Type IV isotherms. According to the definition of the International Union of Pure and Applied Chemistry, the pores are subdivided into micropores (<2 nm), mesopores (2–50 nm), and macropores (>50 nm) [30]. When P/P_o_ < 0.1, a sharp increase in the curve indicated the presence of micropores. A weak hysteresis loop was observed in TC-2 in the range of 0.4–0.9 for P/P_o_, while TC-0.5 and TC-1 curves hadobvious hysteresis, suggesting the existence of abundant mesopores [31]. When the relative pressure P/P_o_ > 0.9, the amount of nitrogen adsorption increased significantly, indicating the existence of macropores. Most of the pores originated from hydrothermal carbon and KOH activation, which provided more active sites and gave the material better electrochemical performance [32]. With the increase of KOH, the specific surface area improved from 25.3 to 2270.6 m^2^ g^−1^. The large specific surface area provided more catalytic active sites for the reaction. Figure 4b reveals that the material had abundant mesopores and a small number of micro-/macropores. The existence of hierarchical pores makes it suitable for supercapacitors with bulky KOH electrolytes. The average pore diameters were 8.05, 4.38, 2.89, and 2.62 nm for the samples, as shown in Table 2. This micro-/mesoporous hierarchical structure could store electrolytes, shorten the electrolyte diffusion distance, and provide electrolytes during charge and discharge.

**Figure 5:**
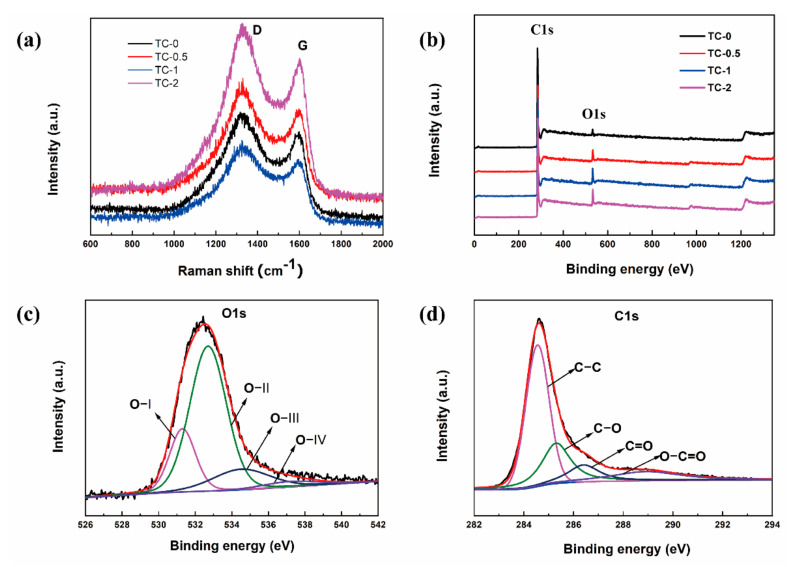
(**a**) Raman spectra and (**b**) XPS survey spectra of TC-0, TC-0.5, TC-1, TC-2. High-resolution (**c**) O1s and (**d**) C1s of TC-1.

The Raman spectra of TC-0, TC-0.5, TC-1, TC-2 are illustrated in Figure 5a. The peak at ~1334 cm^−1^ corresponds to the D band represents the structural defect and disorder. The peak at ~1600 cm^−1^ belongs to the G band, indicating the E_2g_ phonon of sp2 carbons [33]. The intensity ratio (I_D_/I_G_) between the D band and the G band indicates the degree of graphitization. The I_D_/I_G_ of TC-0, TC-0.5, TC-1, TC-2 are 1.26, 1.19, 1.10, 1.03, respectively. It shows an enhancement in graphitization of samples after KOH activation, thereby improving the conductivity of activated carbon.

The surface element content and chemical state of the materials that affect the electrochemical properties were analyzed by XPS. The appearance of two peaks at 284 eV and 532 eV can be seen (Figure 5b), corresponding to C1s and O1s, respectively. The high-resolution C1s (Figure 5c) of TC-1 could be divided into four independent peaks at 284.55 (C–C), 285.3 (C–O), 286.4 (C=O), and 288.9 eV (O–C=O) [34]. O1s (Figure 5d) fitted four basic peaks, located at 531.3, 532.7, 534.4, and 537.2 eV, belonging to C=O (OI), C–OH (O-II), O–C=O groups (O-III), and occluded CO or CO_2_ (O-IV), respectively [35]. The elemental content distribution of TC is also shown in Table 2. The results demonstrate that the TC surface contained large oxygen functional groups. The KOH activation reaction prompted the activated carbon atoms at the edges of the graphite skeleton to trap more oxygen to form functional groups. As the proportion of KOH increased, the content of oxygen atoms increased from 4.72% to 8.90%. The oxygen-containing functional groups improved the wettability of the material, increased the reactive site, and provided a part of the pseudocapacitance behavior [36], thereby giving TC good electrochemical performance. The possible redox reactions of oxygenated functional groups are displayed in (5)–(7) [37] as follows:

>C–OH + OH^−^ ⇌ >CO^−^ + H_2_O (5)

>C=O + OH^−^ ⇌ -COOH+e^−^ (6)

–C=OOH + OH^−^ ⇌ –C=O–O^-^ + H_2_O (7)

**Figure 6:**
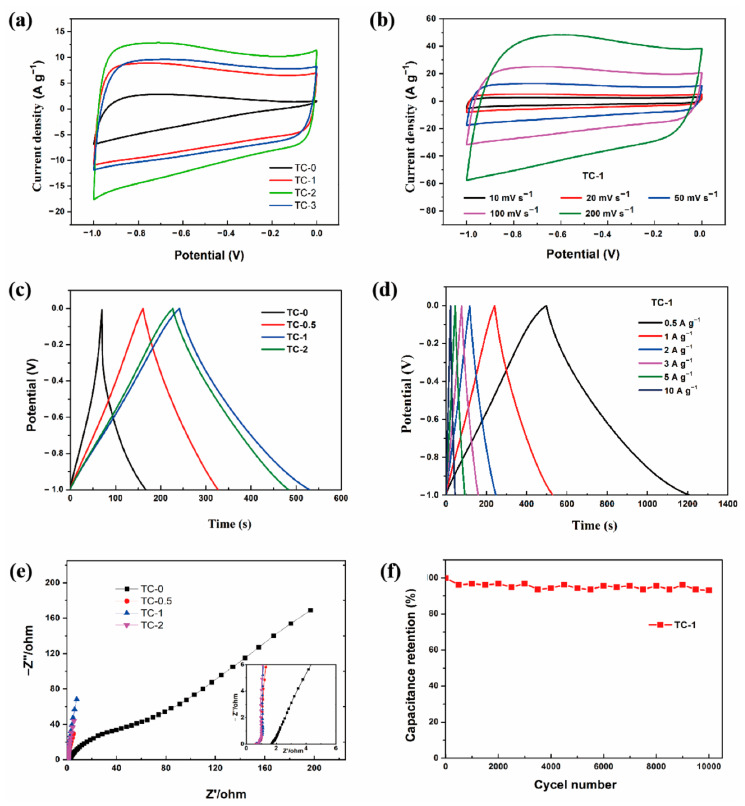
(**a**) Cyclic voltammetry (CV) curves at 50 mV s^−1^, (**c**) galvanostatic charge–discharge (GCD) curves at current density of 1 A g^−1^, (**e**) Nyquist plot of TC-0, TC-0.5, TC-1, and TC-2. (**b**) CV curves at various scan rates, (**d**) GCD curves at various current densities, and (**f**) capacitance retention rate of TC-1 sample in 6M KOH.

The CV curves (Figure 6a) all exhibited quasi-rectangular shapes, which proves that the double-layer capacitance is dominant. The broad hump in the curve is due to the O functional groups, revealing the presence of pseudocapacitance. The maximum integrated area of TC-1 indicates the highest specific capacitance. With the increase of the scanning rate to 200 mV s^−1^ (Figure 6b), the curve still maintained a relatively rectangular shape. It benefited from sufficient micro-/meso-/macropores in the carbon structure, which mitigated the ion diffusion limitation. The results were consistent with the SEM. Figure 6c shows the comparative plots of the TC electrodes at a current density of 1 A g^−1^. A slightly distorted triangular shape was observed, which illustrated that the double electric layer capacitor dominates while containing pseudocapacitance. The specific capacitances of TC-0, TC-0.5, TC-1, and TC-2 were 97.0, 165.8, 288.2, and 256.0 F g^−1^ at a current density of 1 A g^−1^, as calculated from Equation (1). The excellent capacitive behavior of TC-1 could be attributed to the interconnected pore structure and appropriate pore-size distribution in the activated carbon network, which would allow the electrolyte to migrate more efficiently inside the material and thus transmit ions quickly. In addition, the appropriate amounts of the O element provided a redox reaction and improved the affinity of the material. The charge and discharge curves of TC-1 (Figure 6d) at different current densities always retained an isosceles triangular shape. No significant pressure drop was observed when the current density increased to 10 A g^−1^, which could be attributed to small internal series resistance inside the material. At the current densities of 0.5, 1, 2, 3, 5, and 10 A g^−1^, the specific capacitances were 356.4, 288.2, 257.5, 245.8, 234.9, and 221.4 F g^−1^, respectively. These values were similar to the performance of biomass activated carbon reported in other studies, as shown in Table 3. Electrochemical impedance spectroscopy (EIS) was applied to investigate the different ion transport–charge transfer properties in the frequency range of 100 kHz to 10 mHz (Figure 6e). In the low-frequency region, the TC curve was almost parallel to the -Z” axis (except for the TC-0) and exhibited good capacitance behavior. The X-axis intercept represented an equivalent series resistance (of TC-0/0.5/1/2 corresponds to R = 1.72, 0.664, 0.643, 0.709 Ω). The intercept value of TC-1 was the smallest, and the slope of the curve was the largest, which further confirmed its good conductivity and double-layer capacitance characteristics. Moreover, the stability of TC-1 at 20 A g^−1^ current density was determined by 10,000 GCD cycles (Figure 6f). After 10,000 cycles, TC-1 maintained a high capacitance retention rate of 93.06% (202.2 F g^−1^ decreased to 188.2 F g^−1^). It was proved that the material with good cycle stability.

**Figure 7:**
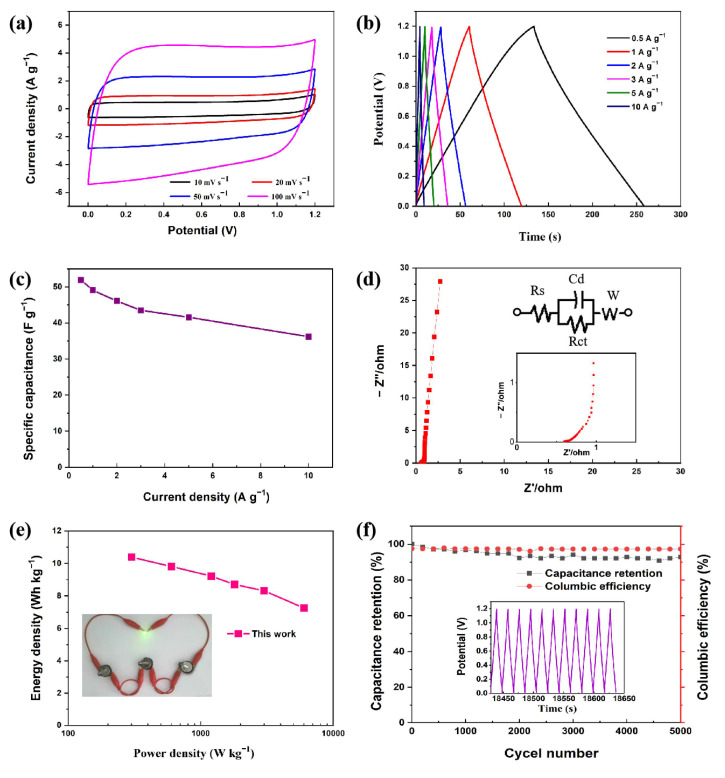
(**a**) CV curves at various scan rates (10–200 mV s^−1^), (**b**) GCD curves at various current densities (0.5–10 A g^−1^), (**c**) specific capacitance curves for different current densities, (**d**) Nyquist plot, (**e**) Ragone plot, and (**f**) cycle stability of the symmetric supercapacitor fabricated by two TC-1 electrodes.

Considering the practical application, we prepared the symmetrical supercapacitor (TC-1//TC-1) by using a TC-1 electrode, and its electrochemical performance was evaluated. The CV curve of TC-1//TC-1 at different scan rates (10–200 mV s^−1^) in the voltage range of 0–1.2 V is presented in Figure 7a. As the scan rate increased, the curve shape remained a standard rectangle, indicating close to the ideal double-layer capacitive behavior [46]. To study the capacitance performance of the TC-1//TC-1 symmetric supercapacitor further, the GCD test was performed at different current densities (Figure 7b). The curves presented a high degree of symmetry without significant voltage drop, revealing the TC-1//TC-1 with small internal resistance and good electrochemical reversibility. At current densities of 0.5, 1, 2, 3, 5, and 10 A g^−1^, the specific capacitances reached 51.9, 49.1, 46.1, 43.5, 41.6, and 36.3 F g^−1^ (Figure 7c), respectively. According to the EIS results, the bulk solution resistance (Rs) and charge transfer resistance (Rct) of TC-1//TC-1 were 0.588 Ω and 0.101 Ω, respectively, which ensured fast charge transfer and ion diffusion. The near-perpendicular line in the low-frequency region conformed to the ideal electric double-layer carbon materials characteristics (Figure 7d) [47]. The Ragone plot of the TC-1//TC-1 symmetrical supercapacitor is shown in Figure 7e. At current densities of 0.5 A g^−1^, the power density was 300 W kg^−1^, and the energy density reached 10.4 Wh kg^−1^, while at current densities of 10 A g^−1^, the power density was 6000 W kg^−1^, and the energy density was 7.3 Wh kg^−1^. The decrease in energy density at high power densities is related to the internal resistance of the material [48]. The power density and energy density of TC-1//TC-1 were comparable or higher than most other biomass carbons reported (Table 3). Furthermore, the cycling performance is an important parameter of the capacitor device. The TC-1//TC-1 symmetric supercapacitor could maintain 92.8% of the initial capacitance and about 97% of coulomb efficiency after 5000 cycles at a current density of 5 A g^−1^, which shows good cycle stability and coulomb efficiency.

## 4. Conclusions

In summary, this study provided ideas for the resource utilization of waste agricultural tobacco stalks and used an environmentally friendly method to transform tobacco stalks into supercapacitor electrode materials. A high-temperature hydrothermal carbon was obtained by using a high-pressure hydrothermal method from the tobacco stalk powder and then was activated with KOH, adjusting the surface chemical composition and porosity by changing the ratio of the activator to achieve good control of its performance. SEM results showed that the high-temperature and high-pressure hydrothermal environment could effectively improve the surface roughness of the material, thereby increasing the specific surface area. The structural analysis confirmed that the TC-2 had a high SSA (2270.6 m^2^ g^−1^) and large porosity. Due to the higher SSA and well-connected micro-/meso-/macropore network, the surface and fast ion transport were fully utilized. The oxygenated groups induce pseudocapacitance and enhance the material wettability, thereby improving the electrochemical properties of the material. The specific capacitance of TC-1 reached 356.4 F g^−1^ when the current density was 0.5 A g^−1^ in the three-electrode test. The symmetrical supercapacitor showed an energy density of 10.4 Wh kg^−1^ at a power density of 300 W kg^−1^. There was still a 92.8% capacitance retention rate after 5000 cycles, and the LEDs were successfully lit. The good electrochemical properties of TC-1 (280 °C, 8 MPa, m(HC):m(KOH) = 1:1) have wide application potential on energy storage devices. An interesting, environmentally friendly, and effective method for the treatment of waste tobacco stalks has proven successful in this paper and should be applicable to recycling other biowaste resources.

## Figures and Tables

**Table 1:** Abbreviations and compositions of tobacco activated carbon specimens investigated.

Abbreviations	HC: KOH (wt.%)
TC-0	1:0
TC-0.5	1:0.5
TC-1	1:1
TC-2	1:2
Blank-TC	No hydrothermal and activation

Abbreviations

HC: KOH (wt.%)

**Table 2:** Textural characteristics and the content of elements of TC.

Samples	BET Surface Area (m^2^ g^−1^)	Pore Volume (cm^3^ g^−1^)	Average Pore Size (nm)	C (wt.%)	O (wt.%)
TC-0	25.3	0.04	8.05	95.28	4.72
TC-0.5	892.4	0.11	4.38	92.55	7.45
TC-1	1875.5	0.25	2.89	91.64	8.31
TC-2	2270.6	0.21	2.62	91.10	8.90

**Table 3:** Comparison of the electrochemical performance of biomass activated carbon reported in this study and other literature.

Carbon Precursor	Pre-Carbonization Condition	Single Electrode			Symmetrical Supercapacitor				Ref.
		Carbon/ KOH	Current Density (A g^−1^)	Specific Capacitance(F g^−1^)	Potential Window (V)	Electrolyte	Power Density (W kg^−1^)	Energy Density (Wh kg^−1^)	
Potatoes	HTC: 200 °C, -MPa	1:1	1	269	0–0.8	6M KOH	405.60	4.27	[38]
Bean sprout	Pyrolysis: 400 °C	1:4	1	421	0–0.8	PVA/KOH	300	5.44	[39]
Metasequoia cone	Pyrolysis: 500 °C	1:3	0.5	326	0–1	6M KOH	129	7.6	[40]
Grape	HTC: 300 °C, -MPa	1:8	0.5	214	0–1.5	6M KOH	4980	12.6	[41]
Fish seed	Pyrolysis: 500 °C	1:3	1	306	0–1.0	6M KOH	125	8.68	[42]
Pinecone	--	5:3	0.5	285	0–1.0	6M KOH	250	6.34	[43]
Areca palm leaves	Pyrolysis: 600 °C	1:2	0.5	141	0–1.5	poly(vinyl alcohol)-Li_2_SO_4_	375	10.3	[44]
Walnut shell	Pyrolysis: 400 °C	1:4	0.5	262.74	0–1.2	6M KOH	180.8	7.97	[45]
Tobacco stalks	HTC: 280 °C, 8MPa	1:1	1	288.4	0–1.2	6M KOH	300	10.4	This work

## Data Availability

The data presented in this study are available on request from the corresponding author.
